# Glycyrrhiza Extract and Curcumin Alleviates the Toxicity of Cadmium via Improving the Antioxidant and Immune Functions of Black Goats

**DOI:** 10.3390/toxics12040284

**Published:** 2024-04-12

**Authors:** Yang Ran, Xiaoyun Shen, Yuanfeng Li

**Affiliations:** 1 College of Life Science and Engineering, Southwest University of Science and Technology, Mianyang 621010, China; yangran@mails.swust.edu.cn (Y.R.); shenxy@swust.edu.cn (X.S.); 2 School of Life Sciences, Liaocheng University, Liaocheng 252000, China

**Keywords:** Guizhou black goat, cadmium, antioxidant capacity, immune function

## Abstract

To investigate the mitigative effects of glycyrrhiza extract (GE) and curcumin (CUR) on the antioxidant and immune functions of the Guizhou black goat exposed to cadmium (Cd), 50 healthy Guizhou black goats (11.08 ± 0.22 kg, male, six months old) were used in a 60-day trial and were randomly assigned to five groups with 10 replicates per group, one goat per replicate. All goats were fed a basal diet, with drinking water and additives varying slightly between groups. Control group: tap water (0.56 μg·L^−1^ Cd); Cd group: drinking water containing Cd (20 mg Cd·kg^−1^·body weight, CdCl_2_·2.5H_2_O); GE group: drinking water containing Cd, at days 31 to 60, the basic diet had added 500 mg·kg^−1^ GE; CUR group: drinking water containing Cd, at days 31 to 60, the basic diet had added 500 mg·kg^−1^ CUR; combined group: drinking water containing Cd, at days 31 to 60, the basic diet had added 500 mg·kg^−1^ GE and CUR. Compared with the Cd group, GE and CUR significantly increased the levels of hemoglobin and red blood cell count in the blood, and the activities of serum antioxidant enzyme activity and immune function in the Guizhou black goat (*p* < 0.05). The treatment effect in the combined group was better than that in the GE and CUR groups. The results showed that GE and CUR improved the antioxidant and immune functions of the serum and livers of the Guizhou black goat and alleviated the toxicity damage of Cd contamination. This research has positive implications for both livestock management and human health.

## 1. Introduction

In recent decades, natural grasslands have been heavily contaminated by heavy metals due to the overexploitation of mineral resources, especially cadmium (Cd) [1,2]. Cd is a toxic mineral that is unnecessary for animal growth, and which is easily accumulated in animals through the food chain and is eventually becoming a serious threat to human health. Trace Cd bioaccumulation does serious harm to humans and animals [3]. The Yunnan–Guizhou Plateau is an important natural habitat for the Guizhou black goat. The local goat industry has been hindered by heavy-metal contamination, and local residents have therefore lost important economic resources [4,5]. Previous studies have demonstrated that applying molybdenum (Mo) fertilizer alone [6] or Mo and sulfur (S) fertilizers [7] onto the polluted natural prairie reduced damage by heavy-metal pollution to animals, but prolonged use may lead to intoxication by minerals in animals. Previous studies found that the deposition of Cd in the liver could lead to liver damage and hepatotoxicity [8]. This type of disease was mainly caused by an imbalance between free radicals and antioxidants, known as oxidative stress [9]. After Cd entered liver cells, it could cause the cells to release pro-inflammatory cytokines (IL-6, TNF-α),and the ROS level in the cells increased sharply, leading to inflammatory damage [10]. Supplementing antioxidants could intervene in the damage caused by environmental pollutants to animals by improving their antioxidant functions [11,12,13], but excessive addition could also cause damage to animals. Generally, Cd can cause damage to cells through a variety of mechanisms such as promoting oxidative stress, inducing apoptosis, triggering DNA damage, interfering with oxidative phosphorylation processes in the mitochondria, and promoting inflammatory responses [14]. These impacts disrupt the internal structure and function of cells, potentially triggering illnesses and pathological alterations at the tissue and organ levels. In terms of environmental contamination, Cd is released into the environment by industrial activities, and its high toxicity causes a variety of negative consequences in the soil, water bodies, and living organisms [15]. Cd is concentrated in living organisms, particularly in aquatic ecosystems, and it spreads through the food chain, eventually impacting human health. Cd pollution in the soil has an impact on crop growth and soil microbial populations, and it has the potential to leak into groundwater and endanger drinking water supplies [16]. Cd pollution in water bodies disrupts aquatic ecosystems and harms the various creatures that live there. All of these present serious health concerns to humans.

Curcumin (CUR) is the main bioactive polyphenol component of the spice turmeric (turmeric), which can strengthen immune function, relieve oxidative stress, and improve the growth of animals [17,18]. Previous studies have demonstrated that CUR up-regulated the oxidative stability of broiler muscle [19] and the anti-inflammatory function of intrauterine growth retardation (IUGR) piglets [20], and also alleviated the Cd toxicity of the liver, kidney, and other organs [17]. Glycyrrhizic acid (GA) is isolated from the roots and rhizomes of glycyrrhiza uralensis, a very well-known ancient herb and a potential source of natural anti-inflammatory agents [21]). In the authoritative medical prescriptions of ancient China, GA was used to treat respiratory tract, stomach, and liver diseases, and also alleviated the toxicity of other drugs. GA has good free radical scavenging and antioxidant effects, enhancing immune function, alleviating oxidative stress, and promoting animal growth [22,23]. Licorice and rhubarb have better protective effects on Cd-induced liver and kidney injuries in rats [24]. However, research on the application of glycyrrhiza extract (GE) and CUR in goats is lacking, and whether they can synergistically alleviate the toxicity of black goats exposed to Cd has not yet been studied. Therefore, this research was assumed to explore the mechanism of GE and CUR in the Guizhou black goat under Cd stress, thereby probing a new avenue to treat early Cd poisoning in the black goat.

## 2. Materials and Methods

### 2.1. The Source of GE, CUR, and Cadmium Chloride

GE (GA ≥ 5%) extracted by ultrafiltration membrane technology was purchased from Xinjiang Longhuiyuan Pharmaceutical Co., Ltd., (Urumqi, China), and CUR extracted by ultrasonication technology was provided by Guangzhou Kehu Biotechnology Co., Ltd., Guangzhou, China (purity ≥ 98%). Cadmium chloride (CdCl_2,_ AR) was obtained from Tianjin Zhiyuan Chemical Reagent Co., Ltd. (Tianjin, China).

### 2.2. Experimental Design

The goat farm was located in Guiyang City, Guizhou Province, China. A total of 50 healthy Guizhou black goats (11.08 ± 0.22 kg, male, six months old) were used in a 60-day trial. All goats were divided into five groups with 10 replicates each, one goat per replicate. Control group: basic diet (0.12 mg·kg^−1^ Cd) and tap water (0.56 μg·L^−1^ Cd); Cd group: basic diet and drinking water containing Cd (20 mg Cd·kg^−1^·body weight, CdCl_2_·2.5H_2_O); GE group: basic diet and drinking water containing Cd, at days 31 to 60, the basic diet with added 500 mg·kg^−1^ GE; CUR group: basic diet and drinking water containing Cd, at days 31 to 60, the basic diet with added 500 mg·kg^−1^ CUR; combined group: basic diet and drinking water containing Cd, at days 31 to 60, the basic diet with added 500 mg·kg^−1^ GE and CUR. GE and CUR were first diluted to 2 kg with a carrier (cornmeal flour), and then added to the goats’ full mixed diet for mixing thoroughly. The drinking water containing Cd was based on the recommended dose of previous studies [25] and the previous investigation results of our teams [26]. Some goats had clinical symptoms such as listlessness, rapid heartbeat, reduced food intake, pale mucosa, and unstable standing. From day 31 to day 60, 30 goats were fed GE and/or CUR diets, respectively. Before the test, the health status of the goats and the quality of feed and drinking water were determined. The results are displayed in Table 1.

### 2.3. Feeding Management

The goats were raised in a sheepfold with a leakage floor. Before the test, the goat sheepfold was thoroughly disinfected with disinfectant, and albendazole was used to expel parasites in the goats. During the trial, the enclosure was cleaned regularly, disinfected once a week, and epidemic prevention procedures were implemented according to the provisions of the sheepfold. The goats were grazed during the daytime, supplemented with concentrate supplement at night, and drank freely. GE and CUR were added to the concentrate supplement [27] (200 g concentrate supplement per goat, including 100 mg additive, once a day at 19:00).

### 2.4. Sample Collection and Analysis of the Blood and Serum

The blood and serum samples were prepared as Li et al. described [28]. The whole blood and serum samples were taken from jugular vein with a vacuum blood collection vessel containing EDTA-K_2_ anticoagulant or separation gel, respectively. The blood samples were stored at 4 °C for analysis. The serum samples were centrifuged (3500 r/min for 10 min), then were stored at −80 °C for further analysis.

The levels of blood hemoglobin (Hb), red blood cell count (RBC), packed cell volume (PCV), and white blood cell count (WBC) were admeasured by automatic blood cell analyzer (SF-3000, Sysmex-Toa Medical Electronics, Kobe, Japan) [29]. The concentrations of serum superoxide dismutase (SOD), glutathione peroxidase (GSH-Px), catalase (CAT), glutathione s-transferase (GST), glutamic-pyruvic transaminase (GPT), glutamic oxaloacetic transaminase (GOT), and the content of serum malondialdehyde (MDA) were admeasured using commercial test kits (Nanjing Jiancheng Bio-Engineering Institute, Nanjing, China) [23]. The ELISA analysis method was used to analyze the concentrations of serum immunoglobulin G (IgG), immunoglobulin M (IgM), immunoglobulin A (IgA), interleukin 6 (IL-6), interleukin 1β (IL-1β), and tumor necrosis factor α(TNF-α) [30]. The concentrations of zinc (Zn), iron (Fe), Cu, and Cd were admeasured using an AA–7000 atomic absorption spectrophotometer (Shimadzu Corporation, Tokyo, Japan) [30].

### 2.5. Liver Sample Collection and Pathological Section Preparation

At the end of the experiment, blood samples were collected from the jugular vein, and the black goats were slaughtered using the electric shock method. A portion of liver tissues (on the same side) were collected and placed in an enzyme free cryopreservation tube, then immediately stored in a liquid nitrogen tank, and transferred to a laboratory −80 °C freezer for storage. Further ipsilateral liver tissues were collected and fixed with 4% paraformaldehyde for analysis. Histological sections of the liver tissue samples were made and observed according to the method introduced by Cui et al. [31]. An appropriate amount of the liver was fixed in 10% neutral formalin, then dehydrated and embedded in paraffin. The pretreated liver samples were serially sectioned according to the standard of 5 μm thickness, routinely stained with hematoxylin and eosin, and observed under a microscope. Histopathological changes of the liver were recorded in detail.

### 2.6. Statistical Analyses 

50 goats were randomly selected from 100 goats (six months old) with similar weights and sex (male) for the experiment, and then randomly assigned to five treatments. SPSS software (SPSS, version 23.0, Inc., Chicago, IL, USA) was used to analyze the data. The ANOVA program and Duncan method were used to analyze for significant differences, and data were presented in the form of mean. *p* ≤ 0.05 indicated a statistically significant difference and *p* > 0.05 indicated no statistical difference.

## 3. Results

### 3.1. The Effect of GE and CUR on the Pathological Structure in the Liver of the Guizhou Black Goat

**Figure 1:**
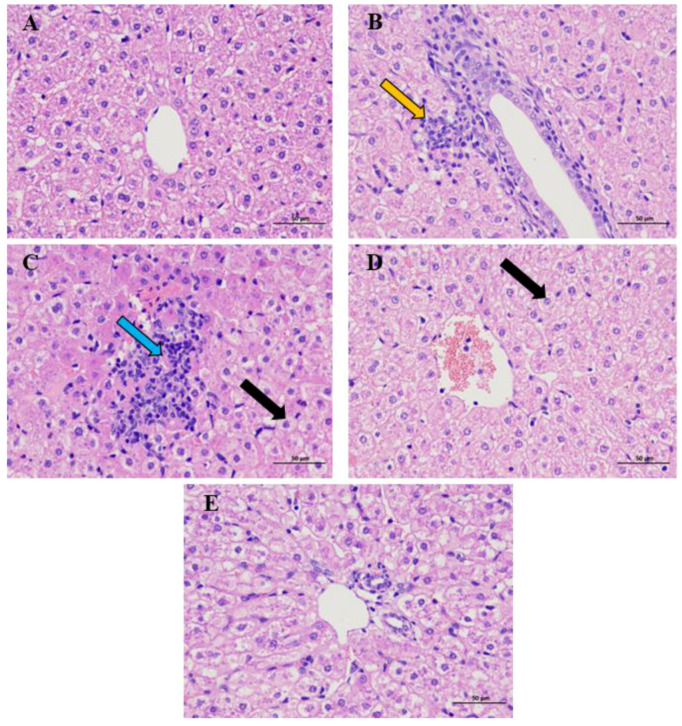
Histopathological changes of the liver in the Guizhou black goat (HE × 400). (**A**) the CON group; (**B**) the Cd group; (**C**) the GE group; (**D**) the CUR group; (**E**) the combined group.

The liver pathological structure under light microscope showed that the arrangement of liver cells of goats from the CON group was tight, the direction of liver cell cords was clear, and the liver sinuses were normal (Figure 1A). Sheet necrosis could be seen in the hepatocytes of the black goat in the Cd group (yellow arrow), with light and no staining in the nuclei, unclear boundaries of liver lobules, and disordered arrangement of hepatocyte cords (Figure 1B). The structure of hepatic lobules in the GE and CUR groups was still clear, the hepatocytes were widely swollen (black arrow), the volume became round, the cytoplasm was loose and lightly stained, and the hepatic sinuses were narrow. A small number of inflammatory cell infiltration foci (blue arrow) could be seen in the liver lobules (Figure 1C,D). The hepatocytes of the black goat in the combined group were closely arranged together with clear cells and outlines and clearly visible liver lobules. Hepatocyte cords were radially arranged around the central vein, and the trend was relatively clear. The hepatic sinuses were narrow, with extensive swelling of hepatocytes, round volume, loose cytoplasm, and light staining. No obvious inflammatory cell infiltration was found (Figure 1E).

### 3.2. The Effect of GE and CUR on the Minerals in the Organ and Tissues of the Guizhou Black Goat

**Figure 2:**
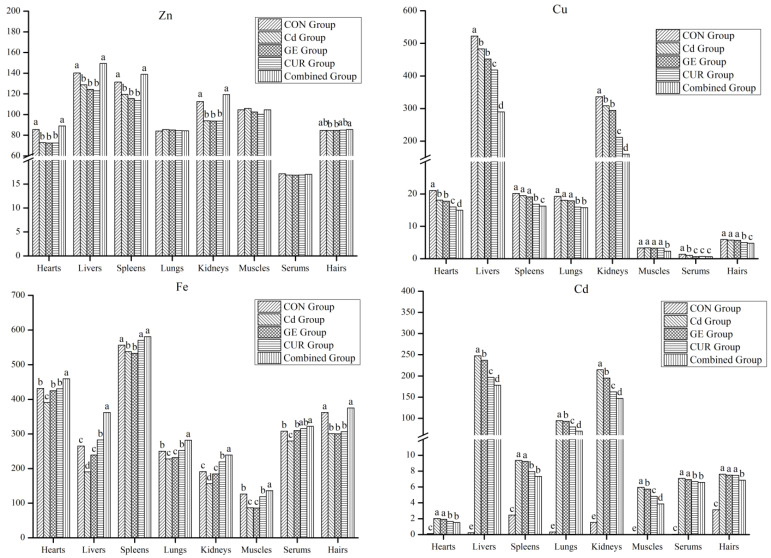
The mineral contents in the organ and tissues of the Guizhou black goat. Zn, zinc; Cu, copper; Fe, iron; Cd, cadmium. The unit of the serum was µg/mL, and the units of other tissues were µg g^−1^. CON Group, control group; Cd Group, cadmium group; GE Group, glycyrrhiza extract group; CUR Group, curcumin group; Combined Group, glycyrrhiza extract + curcumin group. Different small letters of superscript indicate significant difference (*p* < 0.05), and the same small letters or no letters indicate no significant difference (*p* > 0.05).

Compared with the Cd group, the Zn content in the heart, liver, spleen, kidney, and hair of goats from the combined group was significantly up-regulated (*p* < 0.05, Figure 2). The liver and serum Cu content of the black goats from the GE, CUR, and combined groups was extremely increased (*p* < 0.05, Figure 2), the Cu content in the heart, spleen, lung, and muscle of goats from the CUR and combined groups was extremely increased (*p* < 0.05, Figure 2), and the hair Cu level of goats in the combined group was extremely increased (*p* < 0.05, Figure 2). The Fe content in the heart, liver, and kidney of the black goats from the GE, CUR, and combined groups was considerably higher than that in the Cd group (*p* < 0.05, Figure 2), the Fe content in the spleen, lung, and muscle of goats from the CUR and combined groups was extremely increased (*p* < 0.05, Figure 2), and the hair Fe level of goats in the combined group was extremely increased (*p* < 0.05, Figure 2). The Cd content in the liver, lung, kidney, and muscle of the black goat from the GE, CUR, and combined groups was considerably lower than that in the Cd group (*p* < 0.05, Figure 2), the Cd content in the heart, spleen, and serum of goats from the CUR and combined groups was extremely decreased (*p* < 0.05, Figure 2), and the hair Cd level of goats in the combined group was extremely decreased (*p* < 0.05, Figure 2).

### 3.3. The Effect of GE and CUR on the Physiological Indexes in the Blood of the Guizhou Black Goat

The Hb and RBC counts in the blood of goats from the GE, CUR, and combined groups were higher than those in the Cd group (*p* < 0.05, Table 2), and the PCV value in the GE, CUR, and combined groups were greatly up-regulated (*p* < 0.05, Table 2). The WBC count in the blood of goats from the GE, CUR, and combined groups was lower than that in the Cd group (*p* < 0.05, Table 2).

### 3.4. The Effect of GE and CUR on the Antioxidant Capacity in the Serum and Livers of the Guizhou Black Goat

The serum SOD, GSH-Px, and CAT activities in the GE, CUR, and combined groups were higher than those in the Cd group (*p* < 0.05, Table 3), and the serum MDA content in the GE, CUR, and combined groups was lower than that in the Cd group (*p* < 0.05, Table 3). The serum GST, GPT, and GLT activities in the GE, CUR, and combined groups were lower than those in the Cd group (*p* < 0.05, Table 3). The serum SOD activity in the combined group was higher than that in the CON group (*p* < 0.05, Table 3). Compared with the Cd group, the liver SOD, GSH-Px, and CAT activities in the GE, CUR, and combined groups were significantly up-regulated (*p* < 0.05, Table 3), and the liver MDA content of the black goat from the GE, CUR, and combined groups was significantly down-regulated (*p* < 0.05, Table 3). The liver GSH-Px and CAT activities in the combined group were higher than those in the CON group (*p* < 0.05, Table 3).

### 3.5. The Effect of GE and CUR on the Immune Function in the Serum and Livers of the Guizhou Black Goat

Compared with the Cd group, the serum IgG level in the GE, CUR, and combined groups was significantly up-regulated (*p* < 0.05, Table 4), and the serum IgA content in the combined group was greatly up-regulated (*p* < 0.05, Table 4). The concentrations of serum IL-6, IL-1β, and TNF-α in the GE, CUR, and combined groups were lower than those in the Cd group (*p* < 0.05, Table 4). The serum IL-6 concentration in the combined group was lower than that in the CON group (*p* < 0.05, Table 4).

The liver IgG and IgA concentrations in the GE, CUR, and combined groups were significantly higher than those in the Cd group (*p* < 0.05, Table 4), and the levels of liver IL-6, IL-1β, and TNF-α in the GE, CUR, and combined groups were lower than those in the Cd group (*p* < 0.05, Table 4).

## 4. Discussion

### 4.1. The Effect of GE and CUR on the Minerals in the Serum and Livers of the Guizhou Black Goat

An appropriate dose of minerals in the diet can improve the growth of animals, but intake of excessive minerals may cause animal and human intoxication [32,33]. Cu and iron are essential for human and animal growth and are also important components of beneficial enzymes in the body. They are greatly important in animal growth. However, excessive bioaccumulation of Cu in animals may induce oxidative damage [6,34]. Fe is the main component of hemoglobin, various enzymes, and the immune system. Iron metabolism in animals will be interfered with by excessive Cu. Cd is a non-essential toxic trace element, which will interfere with the animal’s metabolism and lead to toxic damage to internal organs [19].

Cd exerts toxic effects on the liver through mechanisms including oxidative stress, inflammatory response, apoptosis, and disruption of lipid metabolism [35]. These mechanisms interact with each other, ultimately leading to liver tissue damage and functional abnormalities. Specifically, in liver cells, Cd can cause oxidative stress, leading to the production of large amounts of ROS, such as superoxide anion, hydrogen peroxide, and hydroxyl radicals. These ROS can damage cellular proteins, DNA, and lipids, resulting in abnormal cell function and cell death. In addition, Cd can activate the inflammatory response in liver cells, generating infiltration of inflammatory cells and the release of inflammatory factors. Prolonged inflammatory response can cause fibrosis and cirrhosis of liver tissue. Furthermore, Cd gives rise to increased apoptosis in liver cells by affecting apoptosis-related signaling pathways and regulating the expression of apoptosis-related proteins. Finally, Cd can interfere with lipid metabolism in liver cells, leading to the accumulation and abnormal distribution of lipids. This can result in fatty liver disease developing and then progressing to non-alcoholic steatohepatitis.

Harmful substances such as Cd can cause toxicity to organs, including the carp spleen [36] and hepatopancreas [37]. Mo fertilizer or Mo and S fertilizers can repair Cd-contaminated soil or reduce the content of heavy metals in the forage [6,7,29], and, therefore, the toxicity to grazing animals of an excessive intake of heavy metals would be avoided. This test found that CUR alone or in combination with GE significantly interfered with the mineral content in the organ and tissues, such as the liver, kidney, serum, or muscle of the Cd-poisoned Guizhou black goat, up-regulated the content of Cu and Fe, and down-regulated the level of Cd. This showed that the combined use of GE and CUR reduced the residue of heavy metal Cd in animal internal organs and reduced the poisonous effects of Cd in the black goat.

### 4.2. The Effect of GE and CUR on the Antioxidant Capacity in the Serum and Livers of the Guizhou Black Goat

The antioxidant system of the animal body is in dynamic equilibrium [38], which is a means of self-protection for animals to deal with environmental stress. A lack of balance between the oxidation and antioxidant systems will cause oxidative damage in animals, thereby inhibiting animal growth [39]. Oxidative damage may cause the accumulation of free radicals in vivo and huge economic losses to animal production [40]. The SOD, GSH-Px, and CAT activities, and the MDA content can reflect the degree of oxidative damage [41]. In vivo, superoxide anions and H_2_O_2_ were removed by SOD, GSH-Px, and CAT [41], which protected cells from damage. Free radicals can also be scavenged by the continuous catalysis of SOD, GSH-Px, and CAT [41]. Previous studies demonstrated that Cd exposure might cause oxidative stress in animals [11]. CUR regulated NF-κB signaling and interleukins secretion in modulating inflammasomes, and reduced the symptoms of heavy metal intoxication [24,42]. Because of the special polyphenol structure of CUR, it can scavenge reactive oxygen species and prevent oxidative damage by combining with free radicals in cells to form stable phenolic molecules [42]. Additionally, it can boost the activity of several antioxidant enzymes, enhancing the antioxidant potential of cells [38]. GE enhanced the antioxidant capacity of weaned piglets and significantly promoted the growth of piglets [43]. GE is high in antioxidants such glycyrrhizic acid and glycyrrhizin, which can neutralize free radicals and reduce cell damage caused by oxidative stress [44]. Additionally, GE possesses anti-inflammatory properties that can lessen the inflammatory response generated by oxidative stress while also directly protecting cells from oxidative damage [44]. Furthermore, GE may stimulate the expression of antioxidant-responsive genes by modulating intracellular signaling pathways such as the Nrf2 signaling pathway, hence increasing cellular resistance to oxidative stress [44]. This study showed that the addition of GE and CUR to the diet of the Guizhou black goat significantly increased serum SOD and GSH-Px activities, and significantly decreased serum MDA content, which might be due to the phenolic hydroxyl with strong reduction ability in GE or CUR scavenging free radicals and inhibiting lipid peroxidation. Compared with the control group, the antioxidant capacity of the Cd group was significantly reduced. Compared with the Cd group, the antioxidant capacity of the GE and CUR groups rebounded. The combined group showed the most significant elevation of antioxidant capacity, and most of the antioxidant indices were significantly different or even better than the control group, such as SOD, GSH-Px, CAT, and MDA. GE and CUR up-regulated the levels of antioxidant enzymes and immune-related factors in the serum and livers of the black goat, thereby reducing the toxicity of Cd. It was suggested that GE and CUR might resist the toxicity of Cd by improving the anti-stress ability of the black goat.

GST exists widely in various tissues and organs of mammals. By catalyzing the coupling of electrophilic groups of certain endogenous or exogenous harmful substances with the thiol group of GSH, its hydrophobicity is increased, making it easy to cross cell membranes and excrete from the body, thereby achieving detoxification and protecting DNA and some proteins from damage [45]. After liver cells are damaged, GST is quickly released into the blood, so an increase in GST in the blood can serve as a sensitive indicator of liver damage [46]. GST has a dual function of clearing peroxides and detoxifying the body. GPT and GLT mainly come from the liver and are important indicators of liver function. When the liver undergoes lesions or losses, their concentrations may be higher [47]. This study found that the levels of GST, GPT, and GLT in the serum of Cd-poisoned black goats significantly increased, but their levels decreased to varying degrees after adding GE and CUR to the goats’ diet, indicating that GE and CUR had an alleviating effect on the toxic damage of Cd to the goat liver.

### 4.3. The Effect of GE and CUR on the Immune Function in the Serum and Livers of the Guizhou Black Goat

The body’s defense ability against foreign pathogenic antigens indicates the level of humoral immunity of animals [48]. Antibodies and immunoglobulins produced by B cells, including IgG, IgM, and IgA, are both important in measuring humoral immunity. Immunoglobulin in the body can synergistically prevent infection and resist the invasion of various bacteria and toxins, in which IgG plays a principal role in immunity. After infection, pathogens may be dissolved by the combination of IgM and its complement [49]. IgA has antibacterial, antiviral, and “barrier” effects in local mucosal immunity [50]. TNF-α, IL-6, and IL-1β, the main inflammatory cytokine in the stress response, can induce other inflammatory mediators [51,52,53]. TNF-α has synergistic biological effects and can aggravate tissue damage [54]. GE can decrease the IL-6 level and increase the IL-2 level of mice [55]. In this study, GE alone or in combination with CUR significantly increased the IgG or IgA concentrations in the serum and livers, and significantly down-regulated the levels of IL-6, IL-1β, and TNF-α concentrations in the serum and livers of the black goat. The changes to inflammatory cytokines in the serum and livers of the black goat showed that immune function was improved. The immune function of the Cd group was significantly lower compared with the control group. The GE and CUR groups had a higher immune function than the Cd group. The combined group showed significant improvement in immune function, with most immunological indicators similar to or better than the control group, including IgG, IgA, and TNF-α. This further showed that the synergistic effect of GE and CUR could enhance the immune function and alleviate intoxication symptoms. This study provided a new way to treat the oxidative damage and immune decline caused by heavy metal Cd poisoning in the Guizhou black goat.

The findings of this study have practical relevance for livestock management, as GE and CUR could be integrated into feed or supplementation regimens to improve the health and welfare of animals exposed to heavy metals. It also reduces farming costs and economic losses for farmers. Furthermore, the usage of these natural substances is consistent with the growing desire for sustainable agriculture techniques. Additionally, the results have significance for human health, as lowering Cd toxicity in livestock could lead to decreased Cd levels in animal products, improving food safety. It also reduces the usage of antibiotics and other medications, decreases antibiotic residues in animals, and mitigates antibiotic pollution in the environment. Overall, the synergistic effects of GE and CUR on antioxidant and immune functions demonstrate their potential to reduce Cd toxicity in domestic animals, with benefits for both livestock management and human health.

This investigation into the effects of GE and CUR on Cd toxicity in Guizhou black goats has a number of limitations. These include the short duration of the trial, which could be addressed by conducting longer-term studies to understand its sustained impact. This study’s application to other species or environmental situations may be limited, highlighting the need for additional research using various animal models and settings. Other limitations include the relatively small sample size, the restricted range of investigated doses and treatment regimens explored, the concentration of individual species, insufficient consideration of potential environmental factors, and the lack of a complete mechanistic understanding of how these additives exert their protective effects. Future studies that address these issues may offer a more thorough knowledge of the potential advantages and restrictions of utilizing GE and CUR to reduce Cd toxicity in livestock.

## 5. Conclusions

This study showed that GE and CUR enhanced the antioxidant and immune functions and reduced Cd bioaccumulation in animal tissues by affecting the mineral metabolism in the Guizhou black goat. GE and CUR have a synergistic effect on the antioxidant and immune functions of the Guizhou black goat exposed to Cd stress and can alleviate the toxic damage of Cd. This has practical implications for livestock management, improving animal health and welfare, while reducing farming costs and improving animal product safety.

## Figures and Tables

**Table 1:** The mineral contents and physiological indexes of the Guizhou black goats before the test.

Items	Serums	Items	Blood
Zn (µg mL^−1^)	16.75 ± 0.42	Hb (g L^−1^)	95.82 ± 4.25
Cu (µg mL^−1^)	0.73 ± 0.16	RBC (10^12^ L^−1^)	9.76 ± 1.18
Fe (µg mL^−1^)	287.28 ± 22.35	PCV (%)	39.75 ± 3.26
Cd (µg mL^−1^)	0.12 ± 0.05	WBC (10^9^ L^−1^)	9.12 ± 0.66

Zn, zinc; Cu, copper; Fe, iron; Cd, cadmium; Hb, hemoglobin; RBC, erythrocyte count; PCV, packed cell volume; WBC, white blood cell count.

**Table 2:** The physiological indexes in the blood of the Guizhou black goats.

Items	CON Group	Cd Group	GE Group	CUR Group	Combined Group	SEM	*p* Value		
							GE	CUR	GE × CUR
Hb (g L^−1^)	110.38 ^a^	91.58 ^b^	109.91 ^a^	110.20 ^a^	111.26 ^a^	1.85	<0.001	<0.001	<0.001
RBC (10^12^ L^−1^)	11.04 ^a^	9.83 ^b^	11.09 ^a^	11.08 ^a^	11.08 ^a^	0.12	<0.001	<0.001	<0.001
PCV (%)	42.71 ^a^	41.00 ^c^	42.28 ^a^	42.05 ^ab^	41.88 ^b^	0.11	<0.001	0.001	<0.001
WBC (10^9^ L^−1^)	8.61 ^b^	8.98 ^a^	8.63 ^b^	8.70 ^b^	8.65 ^b^	0.03	<0.001	0.002	0.001

Hb, hemoglobin; RBC, erythrocyte count; PCV, packed cell volume; WBC, white blood cell count. CON Group, the control group; GE Group, glycyrrhiza extract group; CUR Group, curcumin group; Combined Group, glycyrrhiza extract + curcumin group. Different small letters of superscript indicate significant difference (*p* < 0.05), and the same small letters or no letters indicate no significant difference (*p* > 0.05).

**Table 3:** The antioxidant indexes in the serum and livers of the Guizhou black goat.

Items	CON Group	Cd Group	GE Group	CUR Group	Combined Group	SEM	*p* Value		
							GE	CUR	GE × CUR
Serums									
SOD (IU mL^−1^)	78.62 ^b^	53.47 ^c^	85.24 ^b^	87.46 ^b^	96.66 ^a^	3.25	<0.001	<0.001	<0.001
GSH-Px (IU mL^−1^)	38.90 ^a^	28.67 ^c^	30.97 ^b^	32.07 ^b^	40.64 ^a^	1.02	<0.001	<0.001	<0.001
CAT (IU mL^−1^)	1.58 ^a^	0.96 ^c^	1.35 ^b^	1.42 ^b^	1.63 ^a^	0.05	<0.001	<0.001	0.038
MDA (nmol mL^−1^)	24.28 ^c^	34.40 ^a^	27.18 ^b^	25.91 ^b^	23.27 ^c^	0.91	<0.001	<0.001	<0.001
GST (IU mL^−1^)	8.92 ^b^	9.65 ^a^	8.73 ^b^	8.58 ^b^	8.16 ^c^	0.23	<0.001	<0.001	0.042
GPT (IU L^−1^)	25.31 ^b^	28.35 ^a^	26.28 ^b^	25.93 ^b^	25.62 ^b^	0.19	<0.001	<0.001	0.368
GLT (IU L^−1^)	112.08 ^b^	125.05 ^a^	110.68 ^b^	112.17 ^b^	108.92 ^b^	0.22	<0.001	<0.001	0.273
Livers									
SOD (IU g^−1^)	59.08 ^a^	35.24 ^c^	48.93 ^b^	50.51 ^b^	59.81 ^a^	2.05	<0.001	<0.001	0.128
GSH-Px (IU g^−1^)	25.27 ^b^	20.41 ^c^	25.24 ^b^	26.36 ^b^	30.44 ^a^	0.79	<0.001	<0.001	0.452
CAT (IU g^−1^)	0.65 ^c^	0.63 ^c^	1.09 ^b^	1.20 ^b^	1.38 ^a^	0.06	<0.001	<0.001	0.002
MDA (nmol g^−1^)	4.78 ^b^	6.50 ^a^	4.95 ^b^	4.75 ^b^	4.53 ^b^	0.19	0.001	<0.001	0.008

SOD, superoxide dismutase; GSH-Px, glutathione peroxide; CAT, catalase; MDA, malondialdehyde; GST, glutathione S-transferase; GPT, glutamic-pyruvic transaminase; GOT, glutamic oxaloacetic transaminase. CON Group, the control group; GE Group, glycyrrhiza extract group; CUR Group, curcumin group; Combined Group, glycyrrhiza extract + curcumin group. Different small letters of superscript indicate significant difference (*p* < 0.05), and the same small letters or no letters indicate no significant difference (*p* > 0.05).

**Table 4:** The immune functions in the serum and livers of the Guizhou black goat.

Items	CON Group	Cd Group	GE Group	CUR Group	Combined Group	SEM	*p* Value		
							GE	CUR	GE × CUR
Serums									
IgG (g L^−1^)	36.19 ^ab^	33.19 ^b^	37.83 ^ab^	38.29 ^ab^	39.72 ^a^	0.55	<0.001	<0.001	<0.001
IgM (g L^−1^)	1.68	1.66	1.67	1.69	1.70	0.02	0.762	0.438	0.929
IgA (g L^−1^)	2.03 ^a^	1.65 ^c^	1.91 ^ab^	1.85 ^b^	2.01 ^a^	0.04	<0.001	0.004	0.231
IL-6 (ng L^−1^)	78.07 ^b^	93.07 ^a^	81.64 ^b^	79.43 ^b^	70.11 ^c^	1.87	<0.001	<0.001	0.271
IL-1β (ng L^−1^)	6.24 ^c^	7.54 ^a^	6.63 ^b^	6.47 ^bc^	6.21 ^c^	0.11	<0.001	<0.001	0.001
TNF-α (ng L^−1^)	0.74 ^b^	0.84 ^a^	0.77 ^b^	0.75 ^b^	0.73 ^b^	0.01	0.001	0.007	0.106
Livers									
IgG (g kg^−1^)	42.65 ^a^	34.77 ^c^	41.95 ^b^	42.09 ^ab^	42.30 ^a^	0.68	<0.001	<0.001	<0.001
IgM (g kg^−1^)	18.68	18.64	18.69	18.75	18.79	0.03	0.386	0.071	0.926
IgA (g kg^−1^)	10.85 ^a^	5.47 ^c^	9.51 ^b^	9.97 ^b^	11.05 ^a^	0.47	<0.001	<0.001	<0.001
IL-6 (ng kg^−1^)	0.67 ^b^	0.82 ^a^	0.68 ^b^	0.67 ^b^	0.62 ^b^	0.02	0.001	<0.001	0.082
IL-1β (ng kg^−1^)	6.62 ^bc^	7.74 ^a^	6.96 ^b^	6.67 ^bc^	6.49 ^c^	0.11	<0.001	<0.001	0.011
TNF-α (ng kg^−1^)	5.48 ^c^	7.05 ^a^	6.34 ^b^	6.39 ^b^	5.24 ^c^	0.14	<0.001	<0.001	0.026

IgG, Immunoglobulin G; IgM, Immunoglobulin M; IgA, Immunoglobulin A; IL-6, interleukin 6; IL-1β, interleukin-1β; TNF-α, tumor necrosis factor-alpha. CON Group, the control group; GE Group, glycyrrhiza extract group; CUR Group, curcumin group; Combined Group, glycyrrhiza extract + curcumin group. Different small letters of superscript indicate significant difference (*p* < 0.05), and the same small letters or no letters indicate no significant difference (*p* > 0.05).

## Data Availability

Dataset available on request from the authors.
